# Ethnoterritorial features of paranoid schizophrenia with comorbid dependence on synthetic cannabinoids

**DOI:** 10.1192/j.eurpsy.2025.1296

**Published:** 2025-08-26

**Authors:** N. Bokhan, G. Selivanov

**Affiliations:** 1 Mental Health Research Institute; 2 Siberian State Medical University, Tomsk, Russia; 3 Tomsk State University, Tomsk, Russia, Tomsk; 4 Saint Petersburg University of State Fire Service of Emercom of Russia; 5 Psychiatric Hospital of St. Nicholas the Wonderworker; 6 Rehabilitation Treatment Center “Child Psychiatry”, St. Petersburg, Russian Federation

## Abstract

**Introduction:**

It is indisputable that against the background of the popularity of various chemical addictions among patients with schizophrenia, it is relevant to study the joint clinical and pathomorphological deformation of two nosological phenomena of paranoid schizophrenia and dependence on modern, increasingly popular drugs, synthetic cannabinoids in various ethno-territorial groups. It is important to consider issues of mutual deformation of mental disorders, their progression, behavioral characteristics and adaptation.

**Objectives:**

to study the ethno-territorial features of paranoid schizophrenia comorbid with abuse of synthetic cannabinoids, clinical dynamics, behavior and adaptations.

**Methods:**

follow-up, clinical-psychopathological methods (PANS, SANS, CGI, MMPI, CGI, STAI, LSI, TPA, ICD-10), statistical (Python 3.11.0).

**Results:**

193 men (aged 18 to 35 years) were examined: 142 patients with paranoid schizophrenia dependent on synthetic cannabinoids F20.xx+F12.2xx and 51 - F20.xx without drug addiction. The study took place from 2018 to 2024 on the basis of psychiatric institutions in Russia - Tomsk Region, St. Petersburg, Noyabrsk and Nizhnevartovsk. The phenomenon of abuse of synthetic cannabinoids leads to the development of diseases. Persistent exogenous visual and delirious disorders are included in the complex of symptoms of exacerbation of schizophrenia; A new symptom of pseudohallucinoids appears - thought disorders of an associative (fantasy) disease that arose against the background of long-term exogenous (toxic) effects of the drug on the subject type, usually against the background of a primary endogenous schizophrenic process.

**Conclusions:**

The leading position among patients with schizophrenia who use synthetic cannabinoids in the temperate continental climate zone of Russia was occupied by the following ethnic groups, taking into account the hierarchy: Russians, Tatars; Uzbeks; Germans; Azerbaijanis and Armenians.

**Disclosure of Interest:**

None Declared

